# Characterizing plasma and cerebrospinal fluid biomarkers relevant to neurodegeneration in captive olive baboons (*Papio anubis)*

**DOI:** 10.1371/journal.pone.0318173

**Published:** 2025-02-13

**Authors:** Sarah J. Neal, Sriram Chitta, Elizabeth R. Magden, Joe H. Simmons

**Affiliations:** Department of Comparative Medicine, Michale E. Keeling Center for Comparative Medicine and Research, The University of Texas MD Anderson Cancer Center, Bastrop, Texas, United States of America; Institut d’Investigacions Biomediques de Barcelona, SPAIN

## Abstract

Alzheimer’s disease and related dementias (ADRD) present a significant global disease burden that is only expected to grow in the future. As such, there is a need to develop and investigate biomarkers that identify individuals at risk of developing ADRD with the goal of providing early interventions and treatments. Non-human primate (NHP) models of neurodegeneration present opportunities to examine such biomarkers in a preclinical model with the ability to control several confounding factors present in research with humans. Baboons naturally develop several ADRD-related neuropathologies that humans also exhibit, including age-related tau and amyloid deposition. However, to our knowledge, there are no data characterizing fluid biomarkers relevant to neurodegeneration or ADRD in baboons. We collected plasma (N = 139) and cerebrospinal fluid (CSF, N = 44) from captive baboons ranging in age from 3–19 years old. We characterized biomarkers as a function of age, sex, and rearing status in baboons using a bead-based bioplex human assay (Thermo Fisher Scientific’s Neuroscience 18-Plex Human ProcartaPlex™ Panel). Fluid biomarkers were more detectable in CSF compared to plasma. Additionally, while sex and rearing did not significantly predict biomarkers in baboons, age significantly predicted levels of eight of the 12 biomarkers detected in the assay. Linear regressions showed that CSF levels of total tau, pTau181, NGF-beta, GFAP, NF-H, and S100B were higher in older baboons, as were plasma levels of NGF-beta. Lastly, older baboons showed a higher incidence of co-occurrence of multiple biomarkers as measured in CSF, but not in plasma. These data show that baboons exhibit age-dependent changes in biomarkers used in humans for clinical screening, diagnosis, and prognosis of ADRD, thereby further demonstrating the value of baboons as a model of aging and, possibly, ADRD.

## Introduction

Alzheimer’s Disease and related dementias (ADRD) drastically reduces quality of life for both patients and caregivers, and those with ADRD will succumb to the disease, or complications associated with it [1]. As such, there is a need to develop and investigate biomarkers that identify individuals at risk of developing neurodegenerative pathologies to provide early interventions and treatments. Amyloid β, pTau181, and total tau are the most commonly used cerebrospinal fluid (CSF) biomarkers for ADRD screening, diagnosis, and prognosis, as they have been shown to differentiate among the different ADRD diseases, and correlate with disease severity [1]. Additional biomarkers show promise in screening and diagnostics, such as neurogranin, which reflects axonal damage and synaptic dysfunction, and increases in the early stages of AD [1]. YKL40 indicates neuroinflammation and has been posited as a biomarker that can aid in the differential diagnosis of AD and frontotemporal dementia [2, 3]. Several other biomarkers indicate neuroinflammation (e.g., S100 calcium-binding protein B: S100B, macrophage migration inhibitory factor: MIF, and glial fibrillary acidic protein: GFAP), neurodegeneration (e.g., kallikrein 6: KLK6, Neurofilament light: NfL), and neuronal dysfunction or loss (neural cell adhesion molecule: NCAM, and nerve growth factor: NGF) [3–12].

Blood-based biomarkers (i.e., assayed from serum or plasma) have potential utility in ADRD diagnosis and risk determination. However, research on their utility is still in its infancy, with conflicting findings between known ADRD risk factors and these biomarkers [13, 14], an overall lack of data examining blood-based biomarkers in clinical populations, and the fact that differential levels of blood-based biomarkers may be caused by other factors (e.g., age, sex, comorbidities, concomitant medications, circadian rhythm disruptions) rather than as a function of ADRD risk itself [13]. Indeed, researchers and clinicians currently advise that such blood-based biomarkers should not be used for screening purposes, or if they are, should be used in combination with a full examination of other ADRD risk factors and should be accompanied by cognitive impairment [13, 14]. To increase the utility of these biomarkers for screening, diagnostic, and prognostic purposes, more research is needed to characterize blood-based biomarkers in normal populations, particularly during early and mid-life. Research has demonstrated the trajectories of certain biomarkers across portions of the lifespan using cross-sectional data, such as NfL, which increases with age from birth in normal populations, but other ADRD-relevant biomarker trajectories are unknown. Additionally, data from human populations are highly confounded, often coming from single datapoints rather than longitudinal data, from primarily white populations, and showing increased variability as a function of smoking, disease and BMI status, sleep quality, and more [14].

There is a paucity of animal models that exhibit human-like neurodegenerative pathology, including Aβ deposits, neurofibrillary tangles, general brain atrophy, neuronal loss, and neuroinflammation that occurs in humans with ADRD [15]. Research has shown that non-human primates (NHPs) exhibit certain age-related pathologies that are similar to symptoms of ADRD in humans, including age-related declines in cognition, Aβ deposition, and tau pathology [15–17]. Of all NHPs, chimpanzees are most closely related to humans, and research shows age-related amyloid and tau pathology and impairments in cognition that resemble human mild cognitive impairment [18, 19]. Unfortunately, the scarcity of chimpanzees in research makes investigations of ADRD with this species difficult [18]. In non-ape NHP models, rhesus (*Macaca mulatta*), stump-tailed (*Macaca arctoides*), and cynomolgus macaques (*Macaca fascicularis*), common marmosets (*Callithrix jacchus*), and vervet monkeys (*Chlorocebus aethiops sabaeus*) have been investigated as potential models for ADRD-related neuropathology, and all show some level of age-related decline in cognition as well as the development of Aβ plaques [15, 20]. Additionally, vervet/African green monkeys exhibit lower levels of CSF Aβ with older age [21], and a relationship between lower CSF levels of Aβ42, higher Aβ plaque burden, and negative performance on behavioral measures of AD neuropathological change [22]. CSF levels of Aβ40 and Aβ42 show decreases with age in rhesus and cynomolgus macaques, although tau, pTau181, and NfL showed no relationships with age [20]. Lastly, in aged common marmoset (*Callithrix jacchus*) GFAP, NfL UCH-L1, and total tau levels in serum have been found to be significantly higher than in adults [23]. Together, these studies demonstrate that NHPs provide a useful model for sporadic aging, naturally occurring Aβ deposition, tau pathology, and cognitive impairment [15].

Less research exists examining baboons (*Papio spp*.) as a model for these processes. Baboons reach sexual maturity around 4–5 years of age and live an average of 21 years [24], but can live up 30 years in captivity [25, 26]. According to the National Institutes of Health, baboons are considered geriatric at 15 years of age [27]. Importantly, baboons show certain neurodegenerative hallmarks of ADRD, with changes in brain pathology starting around 20 years of age [28]. One study found age-dependent increases in tau pathology in post-mortem baboon brains, with 71% of the sample aged 21–25 years and 91% of the sample aged 26–30 years showing mild to severe levels of pathology [28]. Another study with post-mortem baboon brains showed increasing Aβ accumulation in the cerebral cortex, hippocampus, and cerebral vessels with age, along with hyperphosphorylated tau in the temporal cortex [29]. Baboons also show microvascular degeneration associated with cerebral amyloid β deposits [30], and age-related cognitive decline [17, 31]. The similarity of baboons to humans in pathology, as well as across social, cognitive, physiological, genetic, and phylogenetic domains, makes baboon models of these diseases highly translational. Indeed, some have noted that baboons may be the best natural animal model for tauopathy given the coexistence of both neuronal and glial tau pathology, age-related progression of such cytoskeletal changes, and selective vulnerability (i.e., susceptibility of particular brain regions to degeneration, including the entorhinal cortex) that is characteristic of Alzheimer’s disease in humans [28, 32]. As such, the captive baboon presents a controlled model to fill some of these knowledge gaps regarding biomarker characterization across the lifespan without the presence of comorbidities or confounds with lifestyle factors in humans. Despite their potential utility, however, there are no data examining age-related differences in blood-based or CSF biomarkers in baboons (*Papio anubis*). Therefore, we aimed to provide the first data for 18 biomarkers associated with ADRD that are indicative of neurodegeneration, neuroinflammation, and/or neuronal loss in captive adult and geriatric baboons. We also aimed to characterize these biomarkers as a function of age, sex, and rearing history (nursery- or mother-reared). Given that nursery-rearing can have a variety of deleterious consequences on behavior, sociality, brain morphology, and immune function, among others [33–42], and is therefore often used as a model for early-life adversity, we hypothesized that nursery-reared baboons would show higher levels of both CSF and plasma biomarkers.

## Methods and materials

Subjects included 139 baboons, ranging in age from 3–19 years (mean age = 8.32, SEM = 0.38; Table 1). Baboons were housed at the Michale E. Keeling Center for Comparative Medicine and Research at The University of Texas MD Anderson Cancer Center in Bastrop, Texas. Baboons were housed in corrals or Primadomes™ with inside access, or in indoor/outdoor runs (Neal et al., 2023) in breeding groups consisting of one or two breeding males, 12–16 breeding females, and their juvenile and infant offspring (0–3 years old). All enclosures are equipped with climbable structure, including multi-tiered platforms, ropes, swings, barrels, culverts, and other manipulanda. Baboons have ad libitum access to water, receive fresh produce twice daily, and are fed a high fiber commercial primate diet (Teklad #7195). The behavioral management program includes the daily provision of environmental enrichment (e.g., destructible items, foraging opportunities, structural enrichment, etc.), as well as positive reinforcement training. The veterinary care program includes biannual physical exams as well as care staff and veterinary health observations three times daily. Data for this study were collected from colony animals during routine biannual physical exams, after which the baboons were placed back into their social groups. Nursery-reared individuals were defined as those baboons that were separated from the dam within 24 hours following birth and cared for by humans, raised in an incubator with access to human infant formula until they were put into small, same-age peer social groups until 2 years-old, when they were introduced to larger adult and sub-adult social groups. Mother-reared individuals were defined as baboons that were not separated from their dam for at least the first 6 months of life and were reared in their natal group.

**Table 1:** Descriptive statistics for CSF and plasma collections across sex and rearing.

	Males			Females		
**Plasma**	**MR**	N	9	**MR**	N	74
		Mean age ± SD	6.8 ± 3.35		Mean age ± SD	9.30 ± 4.87
	**NR**	N	10	**NR**	N	46
		Mean age ± SD	9.07 ± 5.20		Mean age ± SD	9.14 ± 4.72
**CSF**	**MR**	N	5	**MR**	N	18
		Mean age ± SD	12.25 ± 1.71		Mean age ± SD	15.34 ± 3.09
	**NR**	N	5	**NR**	N	16
		Mean age ± SD	11.17 ± 6.39		Mean age ± SD	12.62 ± 4.30

Note: One male baboon had an unknown rearing history

All research and experimental protocols complied with those approved by the UTMDACC Institutional Animal Care and Use Committee (protocol numbers 1665-RN01 and 451-RN00), and complied with the legal requirements of the United States and the ethical guidelines put forth by AALAS, the Animal Welfare Act, The Guide for the Care and Use of Laboratory Animals, and the National Institutes of Health guide for the care and use of Laboratory animals (NIH Publications No. 8023, revised 1978). The Keeling Center has been fully accredited continuously since 1979 by AAALAC.

CSF samples were collected from a subset of 45 baboons (34 females, 23 mother-reared), including 25 geriatric baboons aged 15–19 years (Mean age = 16.41) and 20 nongeriatric adult baboons aged 4–14 years (Mean age = 10.30). Both CSF (cisterna magna puncture) and plasma samples were collected from baboons during routine biannual physical examinations between March 2021 and June 2022. Baboons were immobilized by intramuscular injection of ketamine HCl (5–10 mg/kg body weight) followed by anesthetization with isoflurane inhalation (1–3%) per standard institutional guidelines. We collected 10ml whole blood in EDTA tubes and 2–4 ml of CSF in 15ml polypropylene falcon tubes. Plasma was processed from the whole blood by centrifuging for 10 minutes at a speed of 1,300 x g, then frozen in 0.5 ml aliquots at—80°C within 3 hours of collection. CSF samples were immediately placed on ice, processed and aliquoted into 0.5ml aliquots, then frozen at -80°C within 3 hours of collection. The concentrations of biomarker analytes in CSF and plasma aliquots were measured per the manufacture protocol using a commercially available Neuroscience 18-plex that is commonly used in humans [Thermo Fisher Scientific’s Neuroscience 18-Plex Human ProcartaPlex™ Panel: Amyloid beta 1–42, BDNF, CNTF, FGF-21, GDNF, GFAP, Kallikrein-6 (KLK6), MIF, NCAM-1, Neurogranin, NF-H, NGF beta, S100B, Tau (total), Tau (pTau181), TDP-43, UCHL1, and YKL-40]. On the day of the assay, frozen samples were thawed and precleared by centrifuging at 1400×*g* for 10 minutes at 4C. All incubation and washing steps were performed on a shaker at room temperature. The beads incubated with samples (2h), detection antibody (1h), and SA-PE (30min) were washed after each step, suspended in buffer, and were then acquired on the Biorad Luminex (BioPlex 200) system.

### Data analysis

Biomarker levels are expressed in pg/ml. First, we descriptively summarized distributions of CSF and plasma biomarkers across age groups. We opted to divide baboons into four age groups based on NIH geriatric classification: juvenile: 4 years of age or younger (n = 17; 7 males; 16 mother-reared); young adult: 5–9 years old (n = 57; 11 males; 33 mother-reared); older adult: 10–14 years old (n = 40; 6 males; 16 mother-reared); geriatric: ≥15 years old (n = 25, 4 males; 16 mother-reared) [27]. Levels of each biomarker were divided into quartiles and a score was assigned to each quartile to represent the level of the biomarker present in each individual (0 = absent, 1 = low, 2 = mild, 3 = moderate, and 4 = high).

We then used the raw biomarker values to report mean, standard deviation (SD), median, and range (excluding outliers) for all biomarkers. Given that statistical tests are highly sensitive to outliers, we opted to remove them to examine correlations with demographic parameters using linear regression (see below). Outliers were identified using Tukey’s method, in which observations that fall between the inner and outer fences in each direction are “far out” outliers, while those that fall below the outer fence F1 or above the outer fence F3 are “extreme” outliers (Tukey, 1977). For CSF samples, four baboons had 1–2 outliers, and one baboon had 5 outliers. For plasma samples, 10 baboons had 1–2 outliers, and two baboons had 3 outliers.

We used Pearson’s correlations to examine associations between raw biomarker values within and between CSF and plasma. To examine differences in each biomarker as a function of demographic parameters, we used linear regressions with age entered on the first block of the equation, sex and rearing entered on the second block, and each biomarker with non-zero values as dependent variables. We were specifically interested in examining whether age alone predicted each biomarker, as well as whether sex and rearing predicted biomarkers above and beyond the effect of age. Therefore, when significant, we report the best-fitting model out of the two models, with model 1 being the age only model, and model 2 being the model that includes age+sex+rearing. We report the associated beta, t, and p values for each significant coefficient in the model. The best fitting model was determined using F change statistics in the linear regression, wherein a significant change in the F-value (p≤0.05) denotes that the addition of sex and rearing to the model significantly enhanced the predictive power of the model for that biomarker (i.e., sex and/or rearing uniquely predicted the biomarker level above and beyond the effect of age).

Given the prognostic value of pTau181 to Aβ42 ratio shown in the human literature, we also calculated this ratio to examine relationships with age while controlling for sex and rearing using a linear regression. Lastly, we calculated a plasma and CSF multi-marker score (MMS) for each baboon by counting the number of biomarkers that showed a non-zero value, representing the number of biomarkers detected, in each baboon. We then used the same linear regression approach as described above with the MMS as the dependent variables and age, sex, and rearing as the predictors to examine whether older baboons showed a higher number of biomarkers present in CSF and plasma. All analyses were performed in SPSS (IBM, 2021). The full dataset, including age, sex, rearing, all raw values for all 18 biomarkers across CSF and plasma, for all baboons is available from the Open Science Framework (OSF) data repository: https://osf.io/vxf4w/files/osfstorage/6772cafd9611accaf9116f60.

## Results

**Fig 1:**
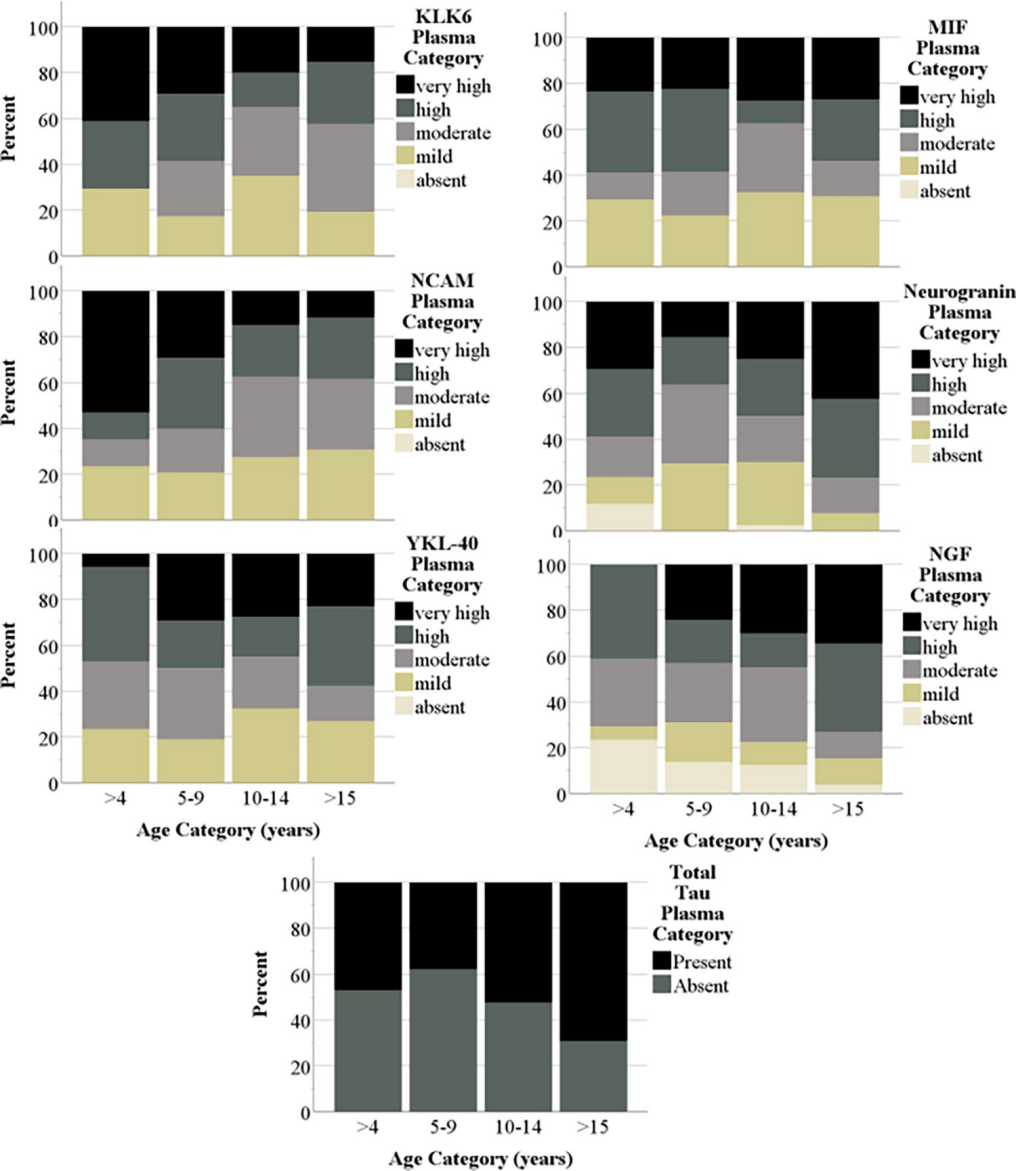
Prevalence of plasma biomarkers across age. Descriptive graphs showing increasing prevalence across plasma biomarkers with age. Levels of each biomarker were divided into quartiles and then a score was assigned to each quartile to represent the level of the biomarker present for each individual (0 = absent, 1 = low, 2 = mild, 3 = moderate, and 4 = high). The plot shows the percent within each age group that exhibits each quartile score. BDNF, CTNF, FGF 21, GDNF, TDP-43, and UCHL1 were not detected in the assay and thus, distributions are not shown here.

**Fig 2:**
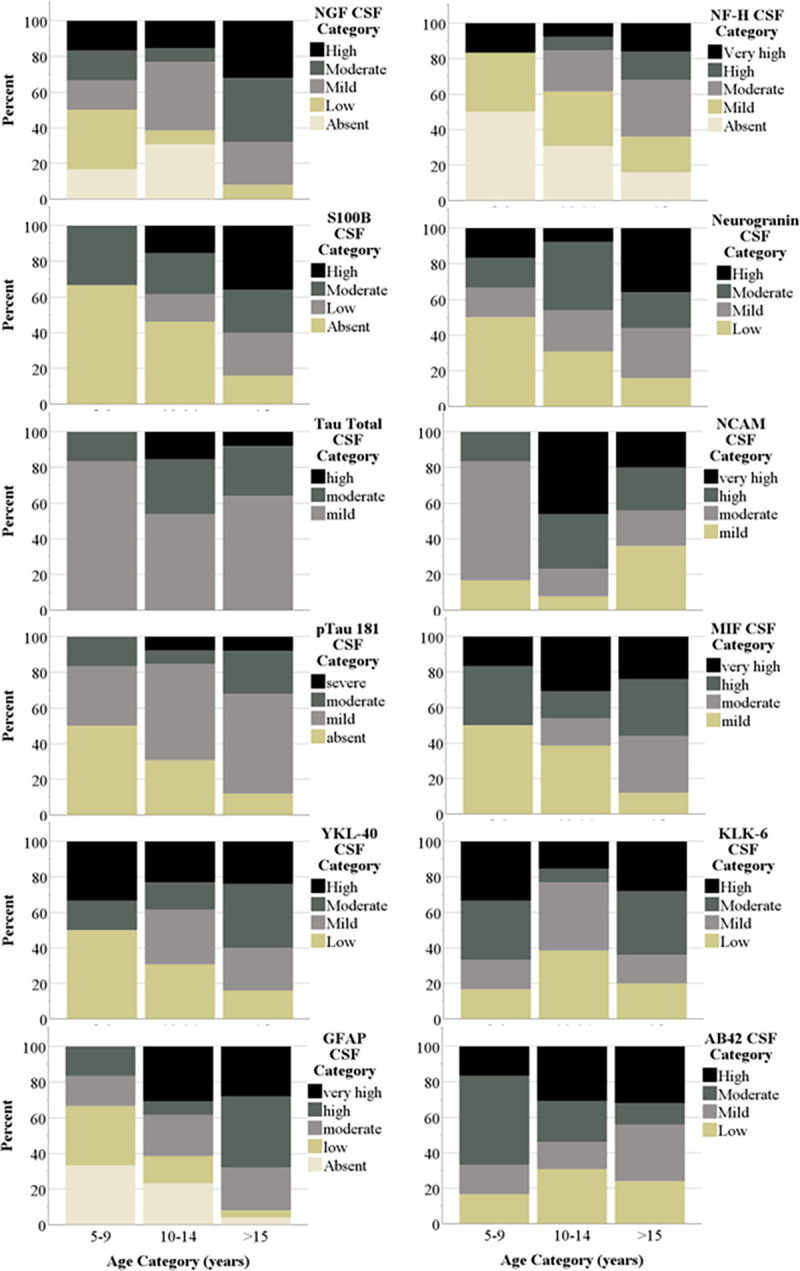
Prevalence of CSF biomarkers across age. Descriptive graphs showing increasing prevalence across CSF biomarkers with age (with the exception of AB42, which shows decreasing prevalence). Levels of each biomarker were divided into quartiles and then a score was assigned to each quartile to represent the level of the biomarker present for each individual (0 = absent, 1 = low, 2 = mild, 3 = moderate, and 4 = high). The plot shows the percent within each age group that exhibits each quartile score. BDNF, CTNF, FGF 21, GDNF, TDP-43, and UCHL1 were not detected in the assay and thus, distributions are not shown here.

Biomarkers were better detected in CSF than plasma, as 7 of the 18 biomarkers in plasma showed non-zero values whereas 12 of the 18 in CSF showed non-zero values (listed as “n/a” in Table 2). More specifically, BDNF, CTNF, FGF 21, GDNF, TDP-43, and UCHL1 were not detected in CSF **or** plasma. Descriptive data showed increasing prevalence with age in ten ADRD biomarkers across the two sample types. Figs 1 and 2 show the percentage of baboons within each age group that exhibits each quartile score in plasma and CSF, respectively. As shown, a higher percentage of older adult and geriatric baboons showed moderate to high levels of several biomarkers compared to juveniles and younger adults.

**Table 2:** Biomarker descriptive data, including outliers removed and resulting sample sizes.

Parameter	Outliers removed from dataset	N	Range	Mean ± SD	Median
***Plasma***					
**Aβ42**	n/a*	141	0–1.47	0.069 ± 0.241	0
**BDNF**	n/a*				
**CNTF**	n/a*				
**FGF 21**	n/a*				
**GDNF**	n/a*				
**GFAP**	n/a*				
**KLK6**	443; 395; 394; 363; 346	136	13.17–336.88	131 ± 71	121.5
**MIF**	110; 74, 71	138	0.67–62.74	16.1 ± 13.5	12.25
**NCAM1**	71776	140	1973–58430	19148 ± 12252	20929
**NG**	2025; 1809; 1644; 1639	137	0–1308	331 ± 330	200
**NF H**	n/a*				
**NGF beta**	45.1; 19.37; 17.73	138	0–11.16	1.0 ± 2.2	0.19
**S100B**	n/a*				
**Tau total**	138; 113, 83, 79; 79	136	0–53	8.3 ± 13.1	0
**pTau181**	n/a*				
**TDP 43**	n/a*				
**UCHL1**	n/a*				
**YKL 40**	8678; 7573; 6762; 6187; 5697	136	91–5107	1503 ± 1116	1159
***CSF***					
**Aβ42**	none	45	2.9–237	236 ± 52	135.4
**BDNF**	n/a*				
**CNTF**	n/a*				
**FGF 21**	n/a*				
**GDNF**	n/a*				
**GFAP**	15052; 7342; 7114; 6511; 2230	40	0–1401.26	231 ± 317	141
**KLK6**	none	45	788–18843	10409 ± 4472	11304
**MIF**	783; 678	43	45–535	155 ± 127	106
**NCAM1**	none	45	22508–59067	36697 ± 8861	36022
**NG**	1473; 756; 717	42	53–493	197 ± 94	177
**NF H**	none	45	0–479	101 ± 130	51
**NGF beta**	none	45	0–1.68	0.397 ± 0.311	0.34
**S100B**	none	45	0–56	8.8 ± 13.4	2.41
**Tau total**	14806; 14331; 12174; 5527	40	32–4058	942 ± 929	738
**pTau181**	48; 37	43	0–26	5.7 ± 6.2	3.83
**TDP 43**	n/a*	45	0–9327		
**UCHL1**	n/a*				
**YKL 40**	none	45	323–15649	4588 ± 3138	4268

Note: Levels are expressed in pg/ml. * indicates that biomarker levels were mostly or all zeros, as such, means could not be calculated. NG: Neurogranin.

Except for NGF-beta and NCAM, all plasma biomarkers showed significant positive relationships with each other, ranging from weak (r = .20 between NGF-beta and total Tau) to very strong (r = .91 between YKL-40 and MIF) (Table 3). CSF biomarker relationships were more varied, with 66.7% of the possible correlations being significant (p<0.05) or trending (p<0.10), and ranging from moderate (r = -.29 between GFAP and YKL-40, p<0.10) to strong (r = .87 between pTau181 and NF-H, p<0.05), including significant negative relationships between MIF and KLK6, as well as YKL-40 and Aβ42. Lastly, correlations between CSF and plasma biomarkers were less strong, ranging from weak (r = .29 between plasma total tau and CSF total tau, p<0.10) to moderate (r = .33 between plasma neurogranin and CSF total tau, p<0.05), with 4.7% of the possible correlations trending, 8.3% significant, and two significant negative relationships (i.e., NGF-beta with KLK-6 and NCAM).

**Table 3:** Pearson’s correlation coefficients between plasma and CSF biomarkers.

Sample type	Bio-marker	Plasma						CSF											
		MIF	NCAM	NG	NGF-beta	Tau Total	YKL-40	AB42	GFAP	KLK-6	MIF	NCAM	NG	NF-H	NGF-beta	S100B	Tau Total	pTau-181	YKL-40
**Plasma**	**KLK-6**	.65**	.70**	.45**	.23**	.22*	.68**	-0.25	-0.30†	-0.22	0.12	0.09	0.04	0.05	-.33*	-0.07	-0.07	-0.18	-0.25
	**MIF**		.62**	.56**	.58**	.29**	.91**	-0.22	-0.24	-0.09	0.06	-0.08	0.07	-0.06	-0.18	-0.09	-0.01	-0.08	-0.06
	**NCAM**			.36**	0.14†	.22*	.60**	-0.18	-0.31†	-0.19	0.06	0.08	0.04	-0.16	-.42**	-0.20	-0.03	-0.19	-0.18
	**NG**				.32**	.54**	.53**	-0.05	-0.05	-0.05	0.15	-0.04	.33*	0.24	0.17	0.26	.33*	0.18	0.03
	**NGF-beta**					.20*	.44**	-0.16	-0.07	0.01	.32*	-0.10	0.26	0.17	0.08	0.07	-0.14	0.15	-0.01
	**Tau Total**						.22*	0.02	0.12	-0.07	.42*	-0.22	.47**	0.14	0.15	0.31†	0.29†	0.13	0.05
	**YKL-40**							-0.12	-0.16	-0.04	-0.07	0.00	0.06	-0.07	-0.19	-0.07	0.05	-0.04	-0.03
**CSF**	**AB42**								.40*	.73**	-.58**	0.12	0.00	0.12	.47**	.33*	.37*	.30*	.69**
	**GFAP**									.33*	-0.08	.32*	.50**	.45**	.50**	.76**	.53**	.50**	0.29†
	**KLK-6**										-.41**	-0.16	0.05	.32*	.62**	.40**	0.30†	.46**	.76**
	**MIF**											-0.10	.38*	0.14	-0.10	0.13	-0.26	0.02	-.32*
	**NCAM**												0.08	-0.11	-0.27	-0.08	0.03	0.01	-0.17
	**NG**													.59**	0.27	.72**	.53**	.55**	0.05
	**NF-H**														.68**	.81**	.57**	.87**	0.25†
	**NGF-beta**															.69**	.40*	.76**	.59**
	**S100B**																.78**	.77**	.50**
	**Tau Total**																	.47**	0.23
	**pTau181**																		.35*

Total N for each correlation between plasma biomarkers ranged from 132–140, between plasma and CSF biomarkers from 36–42, and between CSF biomarkers from 39–45

**Correlation is significant at the 0.01 level.

*Correlation is significant at the 0.05 level.

† Correlation is trending at the 0.10 level. NG: neurogranin.

**Fig 3:**
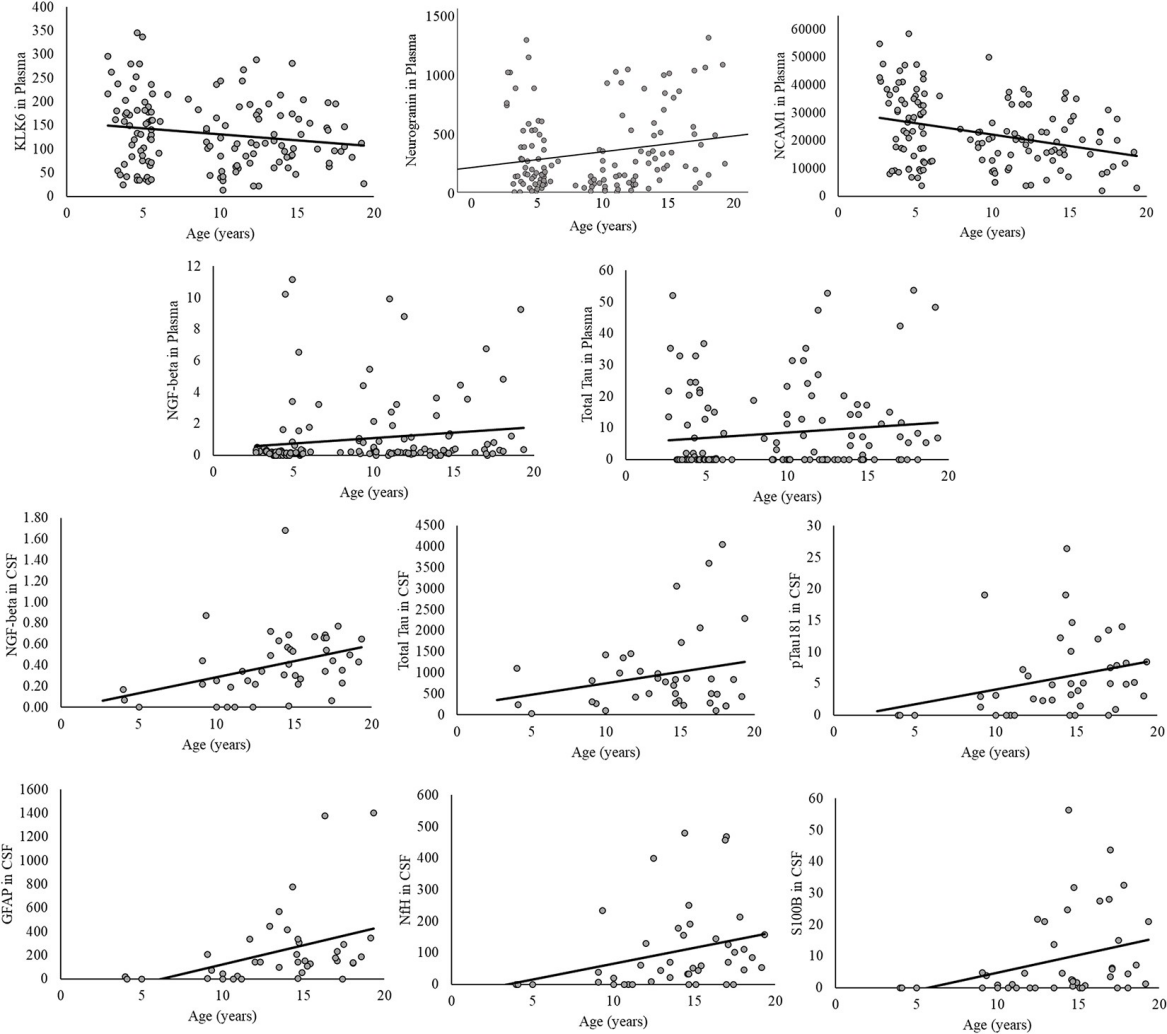
Relationships between age and biomarker levels. Scatterplots showing significant relationships between demographic characteristics and mean biomarker levels (pg/ml) in CSF and plasma.

Linear regressions results are reported in Table 4, along with means and SEM for each biomarker. Biomarkers that showed significant differences as a function of age are plotted in Fig 3 (note that no biomarkers were predicted by sex or rearing). In summary, as measured in plasma, KLK-6, NCAM, neurogranin, and NGF beta were best predicted by age alone. As measured in CSF, Aβ42, MIF, NCAM, neurogranin, KLK-6 and YKL-40 were not predicted by age, sex, nor rearing (p >0.16). CSF levels of GFAP, NF-H, NGF-beta, S100B, total tau, and pTau181 were best predicted by age only.

**Table 4:** Statistical parameters showing differences between individual neurodegenerative biomarkers as a function of demographic parameters.

Sample Type	Biomarker	Best-fitting model*	F change statistics	Overall model	Age	Sex			Rearing		
						Female	Male		MR	NR	
					Sig.	Mean ± SEM	Mean ± SEM	Sig.	Mean ± SEM	Mean ± SEM	Sig.
**Plasma**	**KLK-6**	1	n.s.	F(1,134) = 3.64, p = 0.059, R2adj = .012	beta = -2.50	132 ± 7	139 ± 12	n.s.	138 ± 8	126 ± 10	n.s.
	**MIF**	n.s.	n.s.	n.s.	n.s.	17 ± 1.3	12 ± 1.5	n.s.	16.7 ± 1.4	15.4 ± 1.99	n.s.
	**NCAM**	1	n.s.	F(1,137) = 15.26, p = 0.001, R2adj = .094	beta = -816.04	22052 ± 1088	27644 ± 3000	n.s.	23160 ± 1277	20781 ± 1383	n.s.
	**Neurogranin**	1	n.s.	F(1,134) = 5.47, p = 0.02, R2adj = .03	n.s.	347 ± 32	242 ± 52	n.s.	363 ± 39	285 ± 39	n.s.
	**NGF-beta**	1	n.s.	F(1,135) = 3.43, p = 0.066, R2adj = .018	beta = .071	1.18 ± 0.21	0.19 ± 0.03	n.s.	0.90 ± 0.19	1.26 ± 0.36	n.s.
	**Total Tau**	n.s.	n.s.	n.s.	n.s.	8.71 ± 1.21	5.53 ± 3.0	n.s.	8.5 ± 1.43	8.03 ± 1.8	n.s.
	**YKL-40**	n.s.	n.s.	n.s.	n.s.	1555 ± 109	1217 ± 138	n.s.	1580 ± 120	1400 ± 159	n.s.
	**MMS**	n.s.	n.s.	n.s.	n.s.	6.67 ± 0.11	6.38 ± 0.13	n.s.	6.60 ± 0.09	6.66 ± 0.19	n.s.
**CSF**	**Aβ 42**	n.s.	n.s.	n.s.	n.s.	128 ± 8	122 ± 21	n.s.	135 ± 11	118 ± 11	n.s.
	**GFAP**	1	n.s.	F(1,37) = 6.50, p = 0.02, R2adj = .126	beta = 29.47	249 ± 63	174 ± 53	n.s.	260 ± 72	196 ± 74	n.s.
	**KLK-6**	n.s.	n.s.	n.s.	n.s.	10829 ± 721	9110 ± 1570	n.s.	11071 ± 972	9896 ± 932	n.s.
	**MIF**	n.s.	n.s.	n.s.	n.s.	161 ± 22	136 ± 45	n.s.	147 ± 22	168 ± 34	n.s.
	**NCAM**	n.s.	n.s.	n.s.	n.s.	36116 ± 1467	38492 ± 3005	n.s.	36328 ± 1981	37422 ± 1831	n.s.
	**Neurogranin**	n.s.	n.s.	n.s.	n.s.	199 ± 17	192 ± 32	n.s.	215 ± 22	178 ± 18	n.s.
	**NF-H**	1	n.s.	F(1,42) = 4.59, p = 0.04, R2adj = .077	beta = 10.07	93 ± 20	125± 51	n.s.	115 ± 28	91 ± 28	n.s.
	**NGF-beta**	1	n.s.	F(1,42) = 7.49, p = 0.01, R2adj = .131	beta = 0.03	0.42 ± 0.06	0.31 ± 0.07	n.s.	0.46 ± 0.07	0.32 ± 0.06	n.s.
	**S100B**	1	n.s.	F(1,42) = 5.36, p = 0.026, R2 adj = .092	beta = 1.10	9.28 ± 2.43	7.16 ± 3.24	n.s.	11.60 ± 3.35	6.04 ± 2.05	n.s.
	**Total Tau**	1	n.s.	F(1,33) = 2.41, p = 0.129, R2adj = .036	beta = 53.90	960 ± 163	888 ± 338	n.s.	1101 ± 227	809 ± 192	n.s.
	**pTau181**	1	n.s.	F(1,39) = 4.27, p = 0.046, R2adj = .073	beta = 0.46	6.14± 1.14	4.05 ± 1.11	n.s.	6.8 ± 1.41	4.38 ± 1.22	n.s.
	**YKL-40**	n.s.	n.s.	n.s.	n.s.	4744 ± 471	4103 ± 1281	n.s.	5112 ± 727	4159 ± 590	n.s.
	**MMS**	1	n.s.	F(1,42) = 14.26, p = 0.001, R2adj = .24	beta = .30	11.62 ± 0.40	12.10 ± 0.81	n.s.	12.21 ± 0.43	11.14 ± 0.59	n.s.

*Best-fitting model: 1 = Age only; 2 = age + sex + rearing. Best fitting-model based on Fchange statistics. n.s. = not significant at the p>0.08 level. Biomarker levels are expressed in pg/ml.

**Fig 4:**
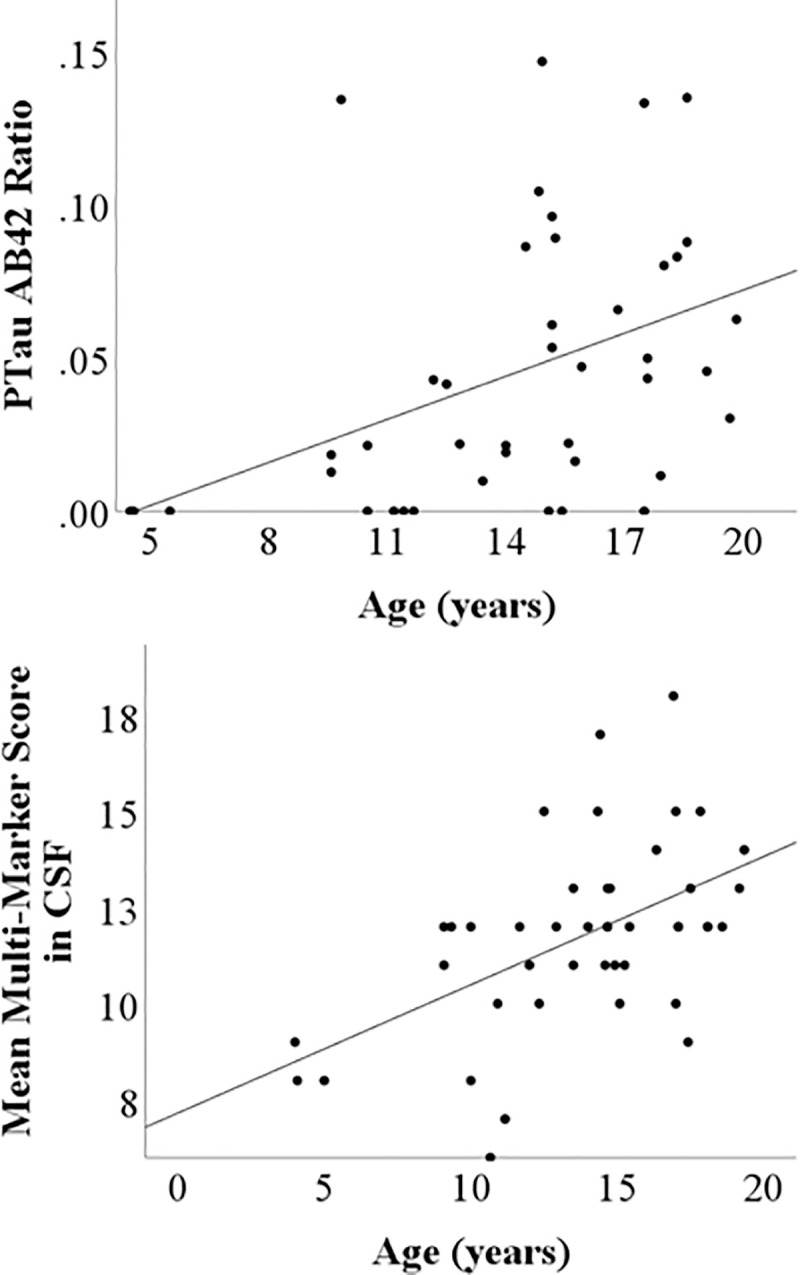
Relationships between age and Ptau181:AB42 and age and multimarker scores. Top Panel: Scatterplot showing relationship between age and phosphorylated tau to AB42 ratio in CSF (pg/ml). Bottom panel: Age significantly predicted CSF multi-marker scores (MMS) such that older baboons exhibited a higher number of ADRD biomarkers present in CSF.

The pTau181 to Aβ42 ratio was significantly predicted by age [F(3,38) = 3.08, p = 0.039, R2adj = .132] while controlling for sex and rearing [age beta = 0.004, t = 2.35, p = 0.024; R2change = .117, F(1,38) = 5.51, p = 0.024, Fig 3]. Lastly, the linear regression examining age, sex, and rearing as predictors of neurodegeneration-related multi-marker scores (MMS) was significant, F(1,42) = 14.26, p<0.001, R2adj = .24. For every three additional years of life, one additional biomarker was present in CSF (beta = .30, t = 3.78, p<0.001; Fig 4). However, the linear regression predicting plasma MMS with age was not significant, p = 0.37.

## Discussion

We provide the most comprehensive examination of fluid neurodegenerative biomarkers in a NHP to date, with biomarkers examined in a relatively large sample in both plasma and CSF, using a clinically relevant 18 biomarker neurodegeneration panel. These data show 1) increasing prevalence of baboon biomarkers used in humans for research as well as clinical screening, diagnostic, and prognostic purposes with advancing age; 2) significant correlations between plasma biomarkers, between CSF biomarkers, and between certain CSF and plasma biomarkers; and 3) that older baboons show a higher number of CSF, but not plasma, biomarkers, with the detection of one additional biomarker per three years of life (equivalent to approximately 9 human years [17]).

It appears that these biomarkers are more easily detected in CSF compared to plasma (or that certain biomarkers are absent in plasma), as 12 were detected in CSF and 7 in plasma. This is perhaps unsurprising given that these biomarkers indicate various forms of neurodegeneration or injury, and, thus, are likely more prevalent in fluids in the brain compared to circulating plasma in the body. However, the correlations between the CSF and plasma biomarkers were varied. For example, total tau and neurogranin showed moderate positive relationships between CSF and plasma levels, but there is mixed evidence regarding these associations in humans, with some research has showing correlations between CSF and plasma tau as well as neurogranin [43], and others showing no such association [44–46]. KLK-6, MIF, NCAM, NGF-beta, and YKL-40 showed no correlation between CSF and plasma levels in the current study. In humans, YKL-40 plasma and CSF levels are correlated [47], an association we replicated in baboons. Research on blood-based biomarkers of ADRD is still in its infancy [13], and there is a lack of data examining associations between plasma and CSF levels of several biomarkers (e.g., MIF, NCAM, and NGF-beta), making it difficult to interpret our results in the context of published data. As such, more research is needed to elucidate what is being measured by plasma compared to CSF samples of these biomarkers in both NHPs and humans.

Several biomarkers showed associations with other biomarkers within the same sample type. For example, one of the stronger significant positive correlations found in the current study was between CSF levels of pTau181 and NF-H. Neurofilaments (including the heavy chain) are essential in axonal structure and transmission of electrical impulses, whereas tau is essential in the microtubular structure of neurons [6, 10, 48]. Neuroaxonal injury or degeneration causes release of both tau and neurofilaments, and as such, both biomarkers reflect non-specific structural damage (axonal or neuronal) [49]. Interestingly, CSF levels of GFAP showed moderate to strong positive correlations with every other biomarker measured in CSF (except for MIF). Under pathological circumstances, astrocytes become reactive and play a part in a variety of neuroinflammatory changes [50]. As such, GFAP, an astrocyte skeleton protein, shows increased expression under such conditions, including in response to Aβ accumulation in the brain [50]. Therefore, it is perhaps unsurprising that GFAP shows positive correlations with biomarkers that are involved in cleaving blood brain barrier and amyloid precursor proteins [KLK-6, [7, 51]], synaptic modulation and plasticity [NCAM and neurogranin [4, 52]], axonal structure, growth, and transmission [NF-H [6, 10]], neurotrophic factors [NGF, [53]], recruitment of macrophages and migration of glial cells [S100B, [54, 55]], neuronal microtubular structure [tau, [48]], and alteration of glial activation and expression of inflammatory factors [YKL-40, [56]].

Sex and rearing did not predict any plasma or CSF biomarkers. The lack of a sex effect is consistent with reports in common marmosets [23], but inconsistent with sex differences found in macaques [20]. However, age predicted 43% (3 of 7) and 50% (6 of 12) of detected plasma and CSF biomarkers, respectively. Furthermore, descriptive data showing the distribution of biomarker levels as a function of age group (Fig 1) shows the increasing prevalence of biomarkers with increasing age across three plasma and six CSF biomarkers. The positive relationships between age, total tau, pTau181 and GFAP are consistent with previous results from macaques, vervets, and marmosets. However, we report the first associations between NGF-beta, S100B, and NfH, all of which are elevated in ADRD as a function of neurodegeneration, neuronal loss, neuroinflammation, and/or axonal damage. This may be unsurprising given that the number one risk factor for ADRD is age, and that positive relationships between age and certain biomarkers have been found in other NHP species. Regardless, the positive relationships with age found in the current study further demonstrates the utility of the baboon as a model for ADRD.

We also report that several plasma biomarkers were predicted by age, including NGF-beta, and negative relationships with KLK-6 and NCAM. Blood-based biomarkers are a timely topic among ADRD and aging researchers and clinicians. The ease with which blood-based biomarkers can be obtained (compared to CSF samples) is appealing to many, and as such, researchers are advocating for the examination of plasma biomarkers in both normal and pathological populations. However, many are cautioning against their clinical use due to the lack of data regarding the prognostic, diagnostic, and screening utility for ADRD [13, 14]. Indeed, some correlations between blood-based biomarkers and ADRD risk may be an artifact of unrelated physiological factors, such as BMI or chronic kidney disease [14]. The idea that weight may be a confounding factor in our study, particularly regarding the negative associations between age and KLK-6 and NCAM, is not supported by our supplementary analyses (see Supporting Information), but more data are needed to determine whether there are other physiological factors that may better explain the relationship between age and these blood-based biomarkers. Indeed, the fact that the correlations between the CSF and plasma biomarkers were absent or mild supports calls for additional research.

To our knowledge, these are the first values published for baboon plasma and CSF neurodegenerative biomarkers, and the descriptive data can be compared to previously reported data for other NHPs, as well as human control and clinical populations (see S1 Table). Mean CSF Aβ42 levels from the current study are comparable to values reported in cynomolgus macaques, marmosets, and vervets, whereas CSF levels of total tau seem inconsistent with other species. The greater prevalence of tau pathology including neurofibrillary tangles in baboon postmortem brains compared to other catarrhine monkeys [30, 57, 58] may explain these discrepancies and may reflect a species difference in neuropathology. Overall, caution should be taken when comparing biomarker levels across NHP species and with human control and ADRD values since not all of the cellular and morphological aspects of ADRD neurodegeneration are fully modeled in any one NHP species. This may be further reflected by the fact that we did not detect several of the biomarkers included in the panel in either CSF or plasma, including BDNF, CTNF, FGF 21, GDNF, TDP-43, and UCHL1. Additionally, we would expect differences across NHP species, as well as between humans and NHPs, given the phylogenetic, genetic, morphological, social, and life-history differences, and the fact that ADRD seems to be uniquely human. As noted in Robertson et al. (2022), species, age, as well as assay-specific differences may account for discrepancies between human and NHP values, as well as across NHP species. Indeed, even while using the same assay, Robertson et al. (2022) found greatly differing levels of Aβ40 and Aβ42 between rhesus and cynomolgus macaques, two closely related species. As such, comparisons within species may prove to be a more prudent approach.

This study was intended as an exploratory study investigating the prevalence of various biomarkers relevant to neurodegeneration and ADRD in baboons. Therefore, we opted to use the Neuroscience 18-Plex Human ProcartaPlex™ Panel given that it is commercially available for research purposes and comes standard with these 18 targets, although not all of them are used for screening and diagnostic purposes. For example, the AB40 to AB42 ratio and NF light chain have been confirmed to be more discriminative of disease states in clinical settings than AB42 and NF heavy chain [59, 60], as available in the current kit and measured in the current study. As such, we are currently using a customized biomarker kit that includes a more targeted list of parameters, including AB40, to measure in the baboons as they continue to age. It is also worth noting that, although Thermofisher reports on the biomarker kit website that this assay has been validated in NHPs, additional validation of this assay specifically for baboons may be worthwhile, for example, by optimizing the lower limit of detection by evaluating different dilution factors or incubation times. While additional validation would be beneficial, we believe these data serve as qualification of this assay in baboons. The assay targets highly conserved proteins, so the fact that the assay produced values for various biomarkers suggests that these proteins were indeed detected in the samples. However, we cannot necessarily say that the markers that showed values of 0 were truly “0”, as we cannot rule out the possibility that the assay did not detect those proteins in the baboon samples in particular.

In summary, we characterized a variety of CSF and plasma neurodegenerative biomarkers in baboons as a function of age, sex, and rearing, but much more characterization is needed. For example, Frye et al. [61] review the evidence for vervet monkeys as a strong model for late-onset AD, including results showing neuropathology, biomarker prevalence, glucose dysregulation, physical decline, gut dysbiosis, and cognitive decline similar to AD in humans. Our goal is to provide a similar characterization of such traits in baboons to determine the utility of baboons for use in ADRD- and aging-related research. Baboons in the current study ranged in age from 3 to 19 years, and there were 17 baboons that were considered geriatric. Given that the onset of tau pathology seems to be approximately 19 years of age [28], we continue to collect CSF and plasma samples for biomarker analyses to examine longitudinal changes in this sample, and we expect significant changes indicating age-related neuropathology as these baboons continue to age. Additionally, we have plans to obtain both cognitive, neuroanatomical, and neurofunctional data using automated cognitive testing systems and MRI scanning to examine correlations between these biomarkers, brain pathology, and cognitive decline [62, 63]. Much more data are needed both in terms of replication in (older) baboons, as well as across ADRD-related behavioral and physiological characteristics. Additionally, given that our older adult and geriatric samples consisted primarily of females, additional data are needed to examine sex differences in biomarkers using a more equal distribution of males and females. Regardless, in conjunction with previous data showing amyloid and tau pathology in aged baboon brains and age-related physical declines [64], the current data highlight the potential utility of baboons in investigations of neurodegenerative biomarker trajectories, as well as the ways that these biomarkers may change as a function of pregnancy and sex hormones, and certain experimental treatments and interventions.

## Supporting information

S1 Table Published fluid biomarker levels across nonhuman primate species to date, including those from the current study. Fluid biomarker concentrations of neurodegenerative biomarkers in the current study, other NHPs species, where available, human control, and Alzheimer’s Disease patients.
(DOCX)

S1 File Supporting analyses regarding weight as a confounding factor. (DOCX)
