# Supplementary material for: Characterizing plasma and cerebrospinal fluid biomarkers relevant to neurodegeneration in captive olive baboons (Papio anubis)
Source: PLoS One. 2025 Feb 13;20(2):e0318173. doi: 10.1371/journal.pone.0318173 (PMC11825030; doi:10.1371/journal.pone.0318173)
Supplement: S1 Table — Fluid biomarker concentrations of neurodegenerative biomarkers in the current study, other NHPs species, where available, human control, and Alzheimer’s Disease patients. (DOCX) [file pone.0318173.s001.docx]

| S1 Table. Fluid biomarker concentrations of neurodegenerative biomarkers in the current study, other NHPs species, where available, human control, and AD patients. | | | | | | | |
| --- | --- | --- | --- | --- | --- | --- | --- |
| **Parameter** | **Current study N=141** | **Rhesus N=49 [20]** | **Cyno N=33 [20]** | **Marmoset N=24 [23]** | **Vervet N=18 [38]** | **Human control** | **AD patient** |
|  |  |  |  |  |  |  |  |
|  |  |  |  |  |  |  |  |
| **Plasma** | | | | | | | |
| **Aβ42** | 0.069 ± 0.241 |  |  |  |  | 750 ng/ml | 326 ng/ml [32] |
|  |  |  |  |  |  |  |  |
| **GFAP** | Undetected |  |  | 334.29 ± 225.93 |  | 105 pg/ml | 272 pg/ml [39] |
|  |  |  |  |  |  |  |  |
| **NfL** |  |  |  | 202 ± 472.49 |  |  |  |
|  |  |  |  |  |  |  |  |
| **KLK6** | 131 ± 71 |  |  |  |  | 0-4 ng/ml | 2 - 10 ng/ml [10] |
|  |  |  |  |  |  |  |  |
| **MIF** | 16.1 ± 13.5 |  |  |  |  | 0.045 ng/ml | 0.113 ng/ml[9] |
|  |  |  |  |  |  |  |  |
| **NCAM1** | 19148 ± 12252 |  |  |  |  | 1361 ± 450 | 2180 ± 751 [40] |
|  |  |  |  |  |  |  |  |
| **NG** | 331 ± 330 |  |  |  |  | 1.29 (.15 - 3.78) ng/ml | .88 (0.03 - 2.91) ng/ml [33] |
|  |  |  |  |  |  |  |  |
| **NGF beta** | 1.0 ± 2.2 |  |  |  |  | 3.9 pg/ml | 5.4 pg/ml [41] |
|  |  |  |  |  |  |  |  |
| **Tau total** | 8.3 ± 13.1 |  |  | 0.463 ± 1.60 |  | 206 pg/ml | 873 pg/ml [42] |
|  |  |  |  |  |  |  |  |
| **pTau181** | Undetected |  |  |  |  | 46 pg/ml | 121 pg/ml [42] |
| **UCHL1** | Undetected |  |  | 218 ± 623.85 |  |  |  |
|  |  |  |  |  |  |  |  |
| **YKL 40 CHI3L1** | 1503 ± 1116 |  |  |  |  | 112.631 ng/ml [43] |  |
|  |  |  |  |  |  |  |  |
| **CSF** | | | | | | | |
| **Aβ42** | 236 ± 52 | 502 ± 208 | 291.5 ± 133 |  | 120 - 620 * | 592 pg/ml | 311 (124 - 483) pg/ml [44] |
|  |  |  |  |  |  |  |  |
| **Aβ42** |  | 2677 ± 890 | 1659 ± 653 |  |  | 4003-8959 pg/ml [20] |  |
| **GFAP** | 231 ± 317 |  |  |  |  | 5000 pg/ml | 15000 pg/ml [39] |
|  |  |  |  |  |  |  |  |
| **KLK6** | 10409 ± 4472 |  |  |  |  | 150 - 450 ng/ml | 150 - 500 ng/ml [10] |
|  |  |  |  |  |  |  |  |
| **MIF** | 155 ± 127 |  |  |  |  | 5000 – 20000 pg/ml | 10000 – 25000 pg/ml [45] |
|  |  |  |  |  |  |  |  |
| **NCAM1** | 36697 ± 8861 |  |  |  |  | 300-350 ng/ml [46] |  |
|  |  |  |  |  |  |  |  |
| **NG** | 197 ± 94 |  |  |  |  | 196 pg/ml | 463 pg/ml [47] |
|  |  |  |  |  |  |  |  |
| **NF H** | 101 ± 130 |  |  |  |  | 0.02-0.80 ng/ml | 0.19-0.64 ng/ml [44] |
|  |  |  |  |  |  |  |  |
| **S100B** | 8.8 ± 13.4 |  |  |  |  | 390 pg/ml | 600 pg/ml [48] |
|  |  |  |  |  |  |  |  |
| **Tau total** | 942 ± 929 | 273 ± 121.5 | 242.5 ± 92 |  |  | 298 pg/ml (95-587) | 523 pg/ml (257-2600) [44] |
|  |  |  |  |  |  |  |  |
| **pTau181** | 5.7 ± 6.2 | 36 ± 13 | 36.5 ± 31 |  | 12-40 * | 50 (18-90) pg/ml | 73 (45-279) pg/ml [44] |
|  |  |  |  |  |  |  |  |
| **NfL** |  | 474 ± 241 | 278 ± 100 |  |  |  |  |
| **YKL 40 CHI3L1** | 4588 ± 3138 |  |  |  |  | 200 ng/ml | 248 ng/ml [2] |
|  |  |  |  |  |  |  |  |
| Levels expressed in pg/ml except when indicated otherwise. NG = Neurogranin. Dark cells indicate that biomarker was not tested in that publication. *extrapolated from Figures in Latimer et al., 2018 | | | | | | | |
|  |  |  |  |  |  |  |  |
