# Supplementary material for: Characterizing plasma and cerebrospinal fluid biomarkers relevant to neurodegeneration in captive olive baboons (Papio anubis)
Source: PLoS One. 2025 Feb 13;20(2):e0318173. doi: 10.1371/journal.pone.0318173 (PMC11825030; doi:10.1371/journal.pone.0318173)
Supplement: S1 File — (DOCX) [file pone.0318173.s002.docx]

Supplementary Analyses

We ran additional statistical analyses to examine the possibility that weight was a confounding factor for the relationships between blood-based biomarkers and age, particularly between plasma KLK6 and NCAM and age. In our sample, baboons ranged in age from 3 to 19 years old, and age and weight are significantly correlated (older animals tend to weigh more, until those animals are fully grown around age 5 or 6). As such, larger animals, having a higher blood volume, may have lower levels of biomarker as a function of dilution. To examine this, we re-ran the age-prediction linear regressions, but added weight as a predictor variable on the second block of the equation. Results showed that weight did not add uniquely to the equation and was not a significant predictor of the plasma biomarkers (p>0.20), and our previous results showing age as a predictor of KLK6 and NCAM remained significant (p<0.05). We then limited our analyses to only baboons 5 years and older, corresponding to adulthood when baboons are fully grown. Again, weight was not a significant predictor of any of the plasma biomarkers (p>0.20).
